# Autism Spectrum Disorders and Drug Addiction: Common Pathways, Common Molecules, Distinct Disorders?

**DOI:** 10.3389/fnins.2016.00020

**Published:** 2016-02-05

**Authors:** Patrick E. Rothwell

**Affiliations:** Department of Neuroscience, University of MinnesotaMinneapolis, MN, USA

**Keywords:** autism, addiction, striatum, accumbens, synapse, dopamine, medium spiny neuron

## Abstract

Autism spectrum disorders (ASDs) and drug addiction do not share substantial comorbidity or obvious similarities in etiology or symptomatology. It is thus surprising that a number of recent studies implicate overlapping neural circuits and molecular signaling pathways in both disorders. The purpose of this review is to highlight this emerging intersection and consider implications for understanding the pathophysiology of these seemingly distinct disorders. One area of overlap involves neural circuits and neuromodulatory systems in the striatum and basal ganglia, which play an established role in addiction and reward but are increasingly implicated in clinical and preclinical studies of ASDs. A second area of overlap relates to molecules like Fragile X mental retardation protein (FMRP) and methyl CpG-binding protein-2 (MECP2), which are best known for their contribution to the pathogenesis of syndromic ASDs, but have recently been shown to regulate behavioral and neurobiological responses to addictive drug exposure. These shared pathways and molecules point to common dimensions of behavioral dysfunction, including the repetition of behavioral patterns and aberrant reward processing. The synthesis of knowledge gained through parallel investigations of ASDs and addiction may inspire the design of new therapeutic interventions to correct common elements of striatal dysfunction.

## Introduction

Autism spectrum disorders (ASDs) are prevalent and devastating neuropsychiatric conditions with a pathophysiology that remains poorly understood. The high heritability of ASDs has motivated the widespread application of advanced sequencing technology to identify genetic variants associated with these disorders (McCarroll and Hyman, 2013). The resulting data sets have revealed an extremely complex genetic architecture, including many genes that each contribute to a fraction of cases (Chen et al., 2015). To sift through this complexity, a growing number of studies have taken genetic variants identified in human patients with ASDs, and introduced corresponding mutations into the genome of laboratory mice. Mice carrying genetic variants associated with neuropsychiatric disease provide an opportunity to probe brain function in a highly specific fashion, and explore underlying mechanisms in a manner that can inform the rational design of therapeutics (Fuccillo et al., 2016). In terms of modeling complex disorders like ASDs, a significant strength of this approach is the construct validity provided by studying genetic variants in mice with known ASD association in humans (Nestler and Hyman, 2010). Mice carrying ASD-associated genetic mutations also exhibit behavioral phenotypes that map onto primary ASD symptom domains, including reduced social interaction and repetitive patterns of behavior (Silverman et al., 2010), providing an additional degree of face validity. Some of these behavioral phenotypes can be corrected by drugs approved for treatment of ASDs in humans (e.g., Peñagarikano et al., 2011), although the limited number of effective medications precludes more rigorous evaluation of predictive validity.

Many of the genes associated with ASDs play a role in regulating synaptic transmission between neurons (Zoghbi and Bear, 2012), including synaptic cell adhesion molecules like neurexins and neuroligins (Südhof, 2008), as well as postsynaptic scaffolding molecules like SHANK (Jiang and Ehlers, 2013). The generation of mouse lines carrying these ASD-associated genetic mutations has provided opportunities to evaluate changes in synaptic transmission across a variety of brain regions. The emerging synaptic architecture of ASDs is nearly as complex as its genetic architecture, with little consistency when phenotypes are compared across different brain regions or different ASD-associated mutations. For instance, the same genetic mutation can produce distinct functional changes at different synapses (Etherton et al., 2011; Földy et al., 2013), and the same synaptic process can be oppositely affected by different mutations (Auerbach et al., 2011). These perplexing results highlight the importance of winnowing down the essential synaptic circuits that contribute to ASD pathogenesis.

One such critical pathway may involve the striatum and interconnected basal ganglia nuclei, which have been implicated by a number of recent mouse studies, and exhibit functional and structural changes in human patients with ASDs that often correlate with symptom severity (e.g., Sears et al., 1999; Hollander et al., 2005; Rojas et al., 2006; Voelbel et al., 2006; Langen et al., 2009, 2014; Delmonte et al., 2012; Abrams et al., 2013). This growing literature on striatal dysfunction in ASDs has, rather surprisingly, implicated pathways and circuit elements known to play a role in drug addiction. The development and progression of addiction have long been tied to a number of striatal neurochemical systems (e.g., Wise, 1987; Koob and Bloom, 1988; Sarnyai and Kovacs, 1994). More recently, chronic drug exposure has been shown to cause changes in the structure and function of striatal synapses (Russo et al., 2010; Grueter et al., 2012), and these forms of drug-evoked synaptic plasticity contribute to a variety of addiction-related behaviors in rodents. Several recent studies suggest genes and molecules canonically associated with ASDs function in the striatum to regulate drug-evoked synaptic and behavioral plasticity in addiction models - another surprising connection between these seemingly distinct disorders.

The purpose of this article is to review the emerging intersection between ASDs and addiction in the striatum, and consider potential implications for the pathophysiology and treatment of both disorders. Many of the topics covered below relate recent publications in the realm of ASDs to longstanding or established concepts in addiction research, though recent examples of addiction research are included when appropriate. The topics and references are drawn from personal familiarity with both fields of research, as well as manual review of literature searches including autism and addiction, autism and striatum, or autism and each of the various signaling pathways discussed below. The manuscript is organized on the basis of emerging common themes, and thus represents an integrative review of select literature that highlights areas of overlap and potential shared mechanisms, rather than a comprehensive or systematic review of research on either ASDs or addiction (Whittemore et al., 2014).

## Striatal pathways in ASDs

The striatum serves as a gateway to the basal ganglia, receiving synaptic input from numerous cortical, thalamic, and limbic brain regions, and relaying information to downstream processing stations in the basal ganglia (Sesack and Grace, 2010; Nelson and Kreitzer, 2014). In humans and primates, the striatal complex includes the caudate nucleus, the putamen, and a ventral striatal region known as the nucleus accumbens. In rodents, the nucleus accumbens also occupies the ventral portion of striatum, while the caudate and putamen roughly correspond to medial and lateral subregions of the dorsal striatum, respectively (Graybiel, 2008). Of these striatal subregions, the nucleus accumbens is most closely associated with reward-related behavioral functions (Carlezon and Thomas, 2009; Sesack and Grace, 2010). Many of these functions pertain to learning reward-related associations, either in terms of cues that predict delivery of reward (classical/Pavlovian conditioning), or actions that must be completed to obtain reward (operant/instrumental conditioning). In human brain imaging studies, normal reward-related activation of the nucleus accumbens is disturbed in patients with ASDs (e.g., Scott-Van Zeeland et al., 2010; Delmonte et al., 2012; Dichter et al., 2012; Kohls et al., 2013; Richey et al., 2014).

Dorsal striatal subregions also play a role in processing reward, particularly in terms of the movements and actions that must be learned and executed in order to obtain reward (Balleine et al., 2007). A number of important dissociations have been reported in the behavioral functions of dorsomedial and dorsolateral striatum in rodents (Yin and Knowlton, 2006; Balleine and O'Doherty, 2010). The model emerging from these studies suggests that dorsomedial striatum is important for behaviors that are flexible and sensitive to outcome, which is often the case early in learning. As actions are repeated many times and become streamlined and automatic, they also become less sensitive to outcome, and this late stage of learning involves dorsolateral striatum. These inflexible and ingrained patterns of behavior are considered “habitual,” and the process of habit formation could contribute to some of the repetitive and stereotyped routines and rituals observed in patients with ASDs. Indeed, many studies have reported structural and functional alterations in the caudate and putamen of human patients with ASDs (e.g., Sears et al., 1999; Eliez et al., 2001; Levitt et al., 2003; Hollander et al., 2005; Haznedar et al., 2006; Silk et al., 2006; Turner et al., 2006; Voelbel et al., 2006; Langen et al., 2007, 2012; Takarae et al., 2007; Di Martino et al., 2011).

Many robust behavioral assays for striatum-dependent reward processing have been developed in rodents. One example is the place conditioning assay, which involves the association between a rewarding stimulus and a distinct set of contextual cues (e.g., floor texture, wall pattern, or chamber odor). The choice to spend time in the presence of these cues vs. a neutral set of cues is operationally defined as a conditioned place preference (CPP), and most drugs of abuse produce CPP (Tzschentke, 1998). Rodents will also develop CPP for contextual cues associated with social interaction with conspecifics, relative to cues experienced during social isolation (Panksepp and Lahvis, 2007; Trezza et al., 2009). This preference for cues associated with social interactions (i.e., “social CPP”) is likely related to the preference for pair-bonded partners exhibited by monogamous species like prairie voles (Carter et al., 1995) and titi monkeys (Carp et al., 2015). Many of the same striatal pathways contribute to social behavior as well as drug reward, suggesting neurochemical systems that originally evolved to mediate social attachment may be hijacked by drugs of abuse (Insel, 2003; Burkett and Young, 2012). Recent studies of mice carrying ASD-associated genetic mutations point to dysfunction of these same striatal systems, which will be reviewed below in terms of both basic function as well as dysfunction in ASDs.

### Oxytocin

The peptide hormone oxytocin contributes to a myriad of social behaviors across many mammalian species (Anacker and Beery, 2013). The pro-social effects of oxytocin have generated substantial interest in its use as a treatment for social deficits associated with ASDs, and there is also some evidence for genetic polymorphisms in the oxytocin receptor associated with ASDs (reviewed by Yamasue et al., 2012). The monogamous behavior of prairie voles is associated with a high density of oxytocin receptors in the nucleus accumbens, and pharmacological blockade of these receptors prevents pair bond formation (reviewed by Insel and Young, 2001), pointing to a key role for oxytocin signaling in the nucleus accumbens in social behavior.

A recent study by Dölen et al. (2013) found that pharmacological antagonism of oxytocin receptors in the nucleus accumbens also blocks social CPP in mice. This result was somewhat surprising, as mice were previously reported to have a relatively low density of oxytocin receptors in the nucleus accumbens compared to other rodent species (Olazábal and Young, 2006). However, conditional genetic deletion of the oxytocin receptors in the nucleus accumbens itself did not impair social CPP. Instead, the oxytocin receptors that mediate social CPP appeared to be expressed on the axon terminals of serotonergic fibers that originate in the dorsal raphe nucleus, and pharmacological antagonism of serotonin 5HT1B receptors in the nucleus accumbens also blocked social CPP (Dölen et al., 2013).

Synaptic plasticity in the nucleus accumbens may be important for encoding the association between social interaction and contextual cues that leads to social CPP. In acute brain slice preparations, stimulation of either oxytocin or serotonin 5HT1B receptors in the nucleus accumbens produced a long-term depression (LTD) of excitatory synapses onto MSNs (Dölen et al., 2013). This reduction of excitatory synaptic drive was associated with a decrease in the probability of presynaptic glutamate release, and provides a plausible synaptic mechanism that may contribute to social reward. Other forms of nucleus accumbens LTD that involve presynaptic changes in glutamate release are impaired by addictive drug exposure (e.g., Fourgeaud et al., 2004; Grueter et al., 2010). This occlusion could contribute to decrements in social behavior caused by drug exposure, like the impairment of social bonding in prairie voles caused by amphetamine exposure (Liu et al., 2010). This impairment can be reversed by oxytocin administration (Young et al., 2014), and oxytocin can attenuate other behavioral effects of psychostimulant administration (reviewed by Sarnyai and Kovács, 2014), clearly demonstrating an interaction between drug effects and the oxytocin system. However, the neurobiological substrata of social and drug reward are at least partially separable, because pharmacological antagonism of oxytocin receptors does not block cocaine CPP (Dölen et al., 2013).

### Dopamine release

Dopaminergic input to the nucleus accumbens originates from dopamine neurons in the ventral tegmental area of the midbrain, and this “mesolimbic” dopamine pathway is closely tied to motivation, reward, and the development of addiction (for reviews, see Wise, 2004; Berridge, 2007; Salamone and Correa, 2012). Mesolimbic dopamine is also important for social behavior in rodents, including pair bond formation in prairie voles (reviewed by Curtis et al., 2006). In mice, activity of the mesolimbic dopamine pathway corresponds to social behavior in real time, and optogenetic manipulations of this pathway affect social interaction (Gunaydin et al., 2014). Given these critical functions of dopamine in social behavior and reward, it is perhaps not surprising that genetic polymorphisms in dopamine signaling genes are also associated with ASDs (e.g., Comings et al., 1991; Hettinger et al., 2008, 2012; De Krom et al., 2009; Hamilton et al., 2013; Bowton et al., 2014; Staal et al., 2015).

A recent report by Karayannis et al. (2014) examined the function of the mesolimbic dopamine system in mice following genetic deletion of *Cntnap4* (also known as *Caspr4*), the gene encoding contactin-associated protein-like 4 (Cntnap4). Cntnap4 is a transmembrane protein that belongs to the neurexin superfamily of cell adhesion molecules, which interact with presynaptic proteins involved in neurotransmitter release (Spiegel et al., 2002). Genetic mutations in other members of this family of molecules have been previously reported in patients with ASDs, and Karayannis et al. (2014) report several new ASD probands with *CNTNAP4* gene disruptions. In mice, they found that expression of Cntnap4 was enriched in midbrain dopamine neurons, as well as inhibitory interneurons in the cerebral cortex, and therefore examined the release of dopamine and GABA from these populations of brain cells. While the release of GABA from cortical interneurons was substantially reduced in *Cntnap4* mutant mice, the release of dopamine in the nucleus accumbens was significantly increased. These divergent effects on release of two different neurotransmitters further underscore the heterogeneous effects that a single ASD-associated genetic mutation can have on different types of synapses.

*Cntnap4* mutant mice also exhibited a variety of aberrant behavioral phenotypes (Karayannis et al., 2014). Most striking was hair loss on the snout, face, and body, which was caused by over-grooming of offspring by parents carrying the *Cntnap4* mutation. This excessive grooming phenotype was reversed by chronic treatment with haloperidol, a dopamine D2 receptor antagonist, suggesting over-grooming was caused by excessive dopamine signaling. Social behavior was not assessed by Karayannis et al. (2014) in *Cntnap4* mutant mice, though it is disrupted by mutations of *Cntnap2*, another member of this protein family (Peñagarikano et al., 2011; Burkett et al., 2015). It will be fascinating to see whether excessive dopamine signaling in the nucleus accumbens of *Cntnap4* mutant mice alters either social reward or behavioral responses to addictive drug exposure.

### Dopamine-sensitive medium spiny neurons

A variety of dopamine receptors are expressed by different cell types in the striatum (Gerfen and Surmeier, 2011). While a small fraction of striatal cells are interneurons that release acetylcholine or GABA, the vast majority of striatal cells are medium spiny projection neurons (MSNs). MSNs are the principal neurons of the striatum and relay information to downstream processing stations in the basal ganglia, including the substantia nigra pars reticulata and the globus pallidus. In the dorsal striatum, striatonigral and striatopallidal MSNs represent two discrete subpopulations that differ in expression of D1 vs. D2 dopamine receptors, as well as a variety of other properties (Gerfen and Surmeier, 2011). The nucleus accumbens also contains discrete populations of D1-MSNs and D2-MSNs, but D1-MSNs in the nucleus accumbens project to both the ventral mesencephalon and ventral pallidum, whereas D2-MSNs project only to the ventral pallidum (Kupchik et al., 2015).

The ability to identify and manipulate specific MSN subtypes was dramatically advanced by the development of bacterial artificial chromosome (BAC) transgenic mice (Heintz, 2001), which allow cell type-specific expression of fluorescent proteins (Gong et al., 2003; Shuen et al., 2008) as well as Cre recombinase (Gong et al., 2007; Durieux et al., 2009). The application of these tools to research on drug addiction has revealed that activation of D1-MSNs promotes addiction-related behaviors, whereas activation of D2-MSNs tends to inhibit the same behaviors (Lobo et al., 2010; Bock et al., 2013; Pascoli et al., 2014; reviewed by Smith et al., 2013). These divergent effects are consistent with classic models of basal ganglia function, in which the direct pathway formed by D1-MSNs and the indirect pathway formed by D2-MSNs exert opposite influences on overall basal ganglia output (Albin et al., 1989; DeLong, 1990).

Striatal MSNs show enriched expression of genes associated with ASDs (Chang et al., 2015), and recent studies have begun to explore how ASD-associated mutations affect specific MSN subtypes. One study focused on ASD-associated mutations in neuroligin-3 (*Nlgn3*), a synaptic cell adhesion molecule that plays important roles in shaping the functional properties of synaptic transmission. Loss-of-function genetic mutations in *Nlgn3* caused a specific impairment of inhibitory synaptic transmission onto nucleus accumbens D1-MSNs (Rothwell et al., 2014). This reduction of inhibitory synaptic transmission is intriguing because human patients with ASDs have been reported to have decreased GABA receptor binding in the nucleus accumbens (Mendez et al., 2013). In mice, nucleus accumbens D1-MSNs appeared to be selectively vulnerable to genetic deletion of *Nlgn3* because it is expressed at a relatively high level compared to neighboring D2-MSNs, or MSNs in the dorsal striatum. Conditional genetic deletion of *Nlgn3* from nucleus accumbens D1-MSNs also caused the development of a more repetitive motor routine on the accelerating rotarod task (Rothwell et al., 2014). This behavioral phenotype has also been reported in several other mouse lines carrying ASD-associated genetic mutations (Kwon et al., 2006; Etherton et al., 2009; Nakatani et al., 2009), and thus might have some relevance to the repetitive and stereotyped movements and routines associated with ASDs in human patients.

Altered striatal structure and function have also been reported in mice lacking the genes normally found on human chromosome 16p11.2 that are deleted in some human patients with ASDs (Weiss et al., 2008). These 16p11 mutant mice have an increased number of striatal cells expressing markers of D2-MSNs, as well as more MSNs co-expressing both D1 and D2 markers, but no change in the number of D1-MSNs (Portmann et al., 2014). These changes in cell number were associated with a relative enlargement in the size of the nucleus accumbens as well as the globus pallidus, which receives synaptic input from D2-MSNs. In 16p11 mutant mice, D2-MSNs also appeared to send ectopic projections to the medial globus pallidus, which is normally only targeted by D1-MSNs. Nucleus accumbens MSNs of 16p11 mutant mice had altered excitatory synaptic properties, including a presynaptic increase in the probability of glutamate release and changes in postsynaptic glutamate receptor complement. Mutant mice also exhibited behavioral phenotypes that included hyperactivity, circling, and a lack of habituation to novelty.

### Opioids

Striatal opioid systems play an important role in the rewarding aspects of social interaction (Burkett et al., 2011; Trezza et al., 2011; Resendez et al., 2013), as well as the rewarding properties of opiate narcotics and other abused drugs (Befort, 2015). The mu opioid receptor (MOR) is a particularly critical mediator of the rewarding effects of opiates like morphine (Matthes et al., 1996). Juvenile mice lacking the MOR gene (*Oprm1*) exhibit a diminished behavioral response to separation from their mothers, suggesting that interaction with the mother may be less rewarding (Moles et al., 2004). Adult *Oprm1* knockout mice also exhibit other ASD-related behavioral phenotypes, including decreased sociability and stereotyped behaviors (Becker et al., 2014). These behavioral changes were associated with dramatic gene expression changes in the nucleus accumbens and dorsal striatum, including a number of genes involved in excitatory synaptic signaling. One of these genes (*Grm4*) encodes the metabotropic glutamate receptor mGluR4, and administration of an mGluR4 positive allosteric modulator effectively relieved many of the behavioral phenotypes exhibited by MOR knockout mice.

Endogenous opioids may interact with other neuromodulatory systems in the nucleus accumbens. MOR knockout mice exhibit a dramatic decrease in mRNA levels for oxytocin in the NAc (Becker et al., 2014). Conversely, oxytocin receptor levels in the NAc are increased (Gigliucci et al., 2014), which could represent a homeostatic response to compensate for loss of endogenous ligand. Intranasal administration of oxytocin to adult male MOR-KO mice led to an increase in the number of ultrasonic vocalizations emitted during interaction with a female mouse (Gigliucci et al., 2014). This interaction between opioid and oxytocin signaling in the NAc suggests a convergence of multiple signaling pathways that may contribute to ASD pathophysiology.

### Endocannabinoids

Brain cannabinoid receptors are activated by both exogenous and endogenous cannabinoids, and play an important role in drug reward (Befort, 2015). Endogenous cannabinoid systems in the nucleus accumbens also regulate social behavior, as local blockade of endocannabinoid degradation enhances social play (Trezza et al., 2012). Social interaction increases levels of anandamide (an endogenous cannabinoid) in the nucleus accumbens, an effect that is stimulated by oxytocin and may contribute to social reward (Wei et al., 2015). Endocannabinoids are important for regulating synaptic function in a variety of ways, including a key role in many forms of synaptic plasticity. Mice lacking FMRP have an impairment of endocannabinoid-dependent LTD of excitatory synapses in the NAc (Jung et al., 2012). This impairment is due to a mislocalization of diacylglycerol lipase-α (DGL-α), a key enzyme in the biosynthetic pathway for the endocannabinoid 2-arachidonoyl-sn-glycerol (2-AG). In the absence of FMRP, DGL-α is localized farther away from the postsynaptic density and is not robustly activated by stimulation of mGluR5. However, this signaling deficit could be rescued by inhibiting the degradation of 2-AG, leading to a normalization of LTD in the NAc as well as some behavioral phenotypes of FMRP knockout mice. ASD-associated genetic mutations in *Nlgn3* did not appear to affect endocannabinoid-dependent LTD in the nucleus accumbens (Rothwell et al., 2014), though these mutations do alter endocannabinoid signaling in other parts of the brain (Földy et al., 2013). In the dorsal striatum, exaggerated LTD has been reported in mice overexpressing eukaryotic translation initiation factor 4E (Santini et al., 2013), which is encoded by the ASD candidate gene *Eif3e* (Neves-Pereira et al., 2009).

### Pathways to dorsal striatum

The progression of drug abuse is thought to involve a transition in the control of behavior from ventral to dorsal striatum (for a recent review, see Everitt and Robbins, 2016). This transition corresponds with a shift in the motivation to take drugs, which becomes less goal-directed and more compulsive or habitual (i.e., insensitive to outcome), leading to continued use despite adverse consequences. This transition in behavioral control may involve polysynaptic pathways that link ventral striatum with dorsal striatum, as neurons in the nucleus accumbens project to dopaminergic centers in the midbrain that subsequently project to more dorsal striatal subregions (Haber et al., 2000; Ikemoto, 2007). This ascending spiral of connectivity may be engaged over the course of chronic drug use and contribute to habitual patterns of addiction (Belin and Everitt, 2008).

Changes in the nucleus accumbens caused by ASD-associated genetic variants could accelerate the transition of behavioral control to more dorsal striatal subregions, leading to more repetitive, stereotyped, or habitual patterns of behavior. However, changes that directly affect the dorsal striatum may also produce similar behavioral consequences: for example, the exaggeration of dorsal striatal LTD in *Eif3e* mutant mice could potentially affect the process of habit formation (Santini et al., 2013). Dysfunction in the dorsal striatum has also been reported in mice carrying gene deletions of *Shank3* (Peca et al., 2011), which encodes a synaptic scaffolding protein and is disrupted in Phelan-McDermid syndrome (an autism spectrum disorder) as well as idiopathic ASDs (reviewed by Jiang and Ehlers, 2013). These *Shank3* mutant mice exhibit reduced social interaction and excessive self-grooming to the point of skin lesions (Peca et al., 2011). MSNs in the dorsal striatum exhibited abnormal morphology and impaired excitatory synaptic transmission, although it is presently unclear if D1- or D2-MSNs were preferentially affected.

## ASD molecules in addiction

Genes and molecules that have been extensively studied in the realm of ASDs have recently emerged as potential mediators of addiction-related behavior. These include the genes known to cause Rett syndrome (MECP2—Amir et al., 1999) and fragile X syndrome (FMR1—Pieretti et al., 1991), which have been topics of sustained research since their initial identification. MeCP2 and FMRP play important roles in the regulation of gene transcription and translation, respectively. In mice, genetic mutations in *Mecp2* and *Fmr1* cause a variety of deficits in synaptic transmission and plasticity throughout the brain, which are thought to contribute to the ASD-related symptoms frequently observed in patients with these syndromes (Zoghbi and Bear, 2012).

These ASD-associated molecules have been shown to regulate multiple aspects of striatal function. For example, dopamine signaling in the striatum is regulated by both MeCP2 (Su et al., 2015) and FMRP (Wang et al., 2008). Genetic deletion of *Fmr1* also affects synaptic structure, function, and plasticity in the nucleus accumbens (Jung et al., 2012; Smith et al., 2014; Neuhofer et al., 2015) as well as the dorsal striatum (Centonze et al., 2008; Maccarrone et al., 2010). The development of drug addiction is strongly tied to dopamine signaling and synaptic remodeling in the striatum, motivating several recent investigations of how genetic mutations in *Mecp2* and *Fmr1* affect behavioral responses to addictive drugs. These studies focused on common behavioral outcomes in addiction research, including CPP as a measure of drug reward, as well as performance an instrumental response to receive contingent delivery of the drug (i.e., drug self-administration). These studies also examined psychomotor activation following acute drug administration, as well as the psychomotor sensitization that occurs following repeated drug exposure, as these behavioral processes are closely tied to striatal dopamine release and remodeling of striatal circuitry (for reviews, see Vanderschuren and Kalivas, 2000; Grueter et al., 2012).

### Methyl CpG-binding protein-2 (MeCP2)

Psychostimulant administration induces MeCP2 phosphorylation in the nucleus accumbens (Deng et al., 2010, 2014). This effect is not seen in MSNs, but is instead selective for fast-spiking GABAergic interneurons that express parvalbumin and GAD67. Behavioral responses to amphetamine administration are altered by a constitutive hypomorphic mutation in *Mecp2* (Deng et al., 2010), as well as a Ser421Ala mutation in Mecp2 that prevents psychostimulant-induced phosphorylation (Deng et al., 2014). Enhanced behavioral sensitivity to psychostimulants is also observed following restricted knockdown of *Mecp2* expression in the nucleus accumbens. These findings suggest phosphorylation of MeCP2 in the nucleus accumbens plays a role in constraining behavioral responses to psychostimulants. However, the role of MeCP2 may be different in the dorsal striatum, as a separate study found cocaine self-administration was reduced following restricted knockdown of *Mecp2* expression in the dorsal striatum (Im et al., 2010).

### Fragile X mental retardation protein (FMRP)

Constitutive genetic deletion of *Fmr1* did not alter the acute locomotor response to cocaine, but decreased locomotor sensitization over course of repeated cocaine exposure, and also attenuated cocaine CPP (Smith et al., 2014). Conditional genetic deletion of *Fmr1* in the nucleus accumbens also decreased locomotor sensitization, though it did not affect cocaine CPP. Cocaine-evoked changes in the structure and function of excitatory synapses onto nucleus accumbens MSNs were also enhanced in *Fmr1* constitutive knockout mice. These data suggest that FMRP serves as a negative regulator of the structural and functional changes at nucleus accumbens synapses caused by chronic drug exposure, similar to MeCP2 (Deng et al., 2014).

## Outlook and open questions

How do these emerging parallels between ASDs and addiction (Table 1) inform our understanding of the pathophysiology of each disorder in relationship to the striatum? Despite these parallels, these are clearly two distinct disorders, as evidenced not only by distinct symptomatology but also the lack of comorbidity. Nevertheless, ongoing research may benefit from more careful consideration of neurobiological and psychological process that may be commonly affected in both disorders. This approach is reminiscent the Research Domain Criterion (RDoC) initiative by the U.S. National Institute of Mental Health (Insel et al., 2010; Insel and Cuthbert, 2015), which aims to establish a new framework for psychiatric research that moves away from specific clinical diagnoses and toward dimensions or constructs with well-defined neurobiological substrata that may be impaired across multiple mental disorders. Below, I consider two behavioral dimensions that may be commonly affected in both ASDs and addiction, as well as potential neural mechanisms and therapeutic implications raised by the shared and distinct elements of these disorders.

**Table 1:** **Striatal signaling pathways and molecules commonly implicated in both addiction and autism spectrum disorders (ASDs); see text for details**.

**Pathway/Molecule**	**Relevance to addiction**	**Relevance to ASDs**
Oxytocin	Attenuates behavioral effects of psychostimulant administration (Sarnyai and Kovács, 2014)	Social reward involves actions of oxytocin in the nucleus accumbens (Dölen et al., 2013)
Dopamine	Important for reward and attribution of incentive salience (Wise, 2004; Berridge, 2007)	Increased release in *Cntnap4* mutant mice (Karayannis et al., 2014)
Opioids	Contribute to rewarding effects of exogenous opiates and other drugs (Befort, 2015)	Autism-related behavioral phenotypes in *Oprm1* mutant mice (Moles et al., 2004; Becker et al., 2014)
Endocannabinoids	Contribute to drug reward and synaptic plasticity (Befort, 2015)	Altered striatal synaptic plasticity in *Fmr1* and *Eif3e* mutant mice (Jung et al., 2012; Santini et al., 2013)
Methyl CpG-Binding Protein-2 (MECP2)	Altered behavioral responses to psychostimulant administration (Deng et al., 2010, 2014; Im et al., 2010)	Deletions cause Rett syndrome (Amir et al., 1999)
Fragile X Mental Retardation Protein (FMRP)	Altered behavioral and synaptic responses to cocaine exposure (Smith et al., 2014)	Deletions cause fragile X syndrome (Pieretti et al., 1991)

### Repetitive behavioral patterns as a common dimension of dysfunction

One hallmark of both ASDs and addiction is the repetition of specific patterns of behavior, sometimes in the absence of an obvious goal or even in the face of adverse consequences. Indeed, the transition from casual to compulsive or habitual drug use is one of the defining features of addiction. Conversely, ASDs are associated with behavioral patterns that are performed in a stereotyped fashion and are resistant the change. This can include simple movements like hand flapping, as well as more complex routines and rituals. While somewhat different terminology is used to describe these behavioral patterns (e.g., “compulsive” or “habitual” for addiction, “repetitive” or “stereotyped” for ASDs), all may be characterized by a failure to inhibit the repetition of behavioral patterns. This lack of inhibitory control over repetitive behavior may also contribute to other mental disorders, such as obsessive-compulsive disorder, which is also linked to striatal dysfunction (Burguière et al., 2015).

Rodent models of ASDs and addiction obviously lack the nuance and specificity of the clinical condition, but some interesting similarities in repetitive behavioral patterns seem to suggest common striatal circuitry may be affected in both disorders. For example, stereotyped patterns of rotational behavior caused by ASD-associated genetic mutations (Portmann et al., 2014; Rothwell et al., 2014) are also observed following exposure to psychostimulants (Fowler et al., 2001). Similarly, the orofacial stereotypy caused by repeated exposure to a high dose of cocaine is exacerbated in *Fmr1* knockout mice (Smith et al., 2014). While it is obviously difficult to extrapolate these simple rodent behaviors to the symptoms of complex human disorders likes ASDs and addiction, these examples at least illustrate that some elementary forms of repetitive behavior can be similarly affected by addictive drugs and ASD-associated genetic mutations.

### Aberrant reward processing as a common dimension of dysfunction

The processing of reward-related information represents another behavioral dimension that may be affected in both ASDs and addiction. The concept of reward figures prominently in addiction, where decades of research have led to the identification and dissection of distinct components of reward (reviewed by Berridge et al., 2009). One important distinction is between the hedonic impact or “liking” of reward, and the incentive salience or “wanting” associated with rewards and related environmental stimuli. Repeated drug exposure is thought to selectively exaggerate or sensitize the incentive salience attributed to drugs and drug-related cues, a process that is closely linked to dopamine transmission (for a recent review, see Robinson and Berridge, 2008).

In ASD research, aberrant “reward processing” often refers to altered patterns of brain activity in response to social stimuli or monetary reward (e.g., Scott-Van Zeeland et al., 2010; Delmonte et al., 2012; Dichter et al., 2012; Kohls et al., 2013; Richey et al., 2014). Reduced sensitivity to social stimuli may contribute to the deficits in social behavior associated with ASDs, but the precise nature of this aberrant reward processing remains unclear. For instance, are social deficits in ASDs due to decreased hedonic impact or “liking” of social interaction, or rather to a decrease in the incentive salience or “wanting” associated with social stimuli? An alternative possibility (discussed in more detail below) is that ASDs are associated with indiscriminate attribution of incentive salience to inappropriate stimuli in the environment, which could lead to fixation on these stimuli at the expense of social interaction. Rodents carrying ASD-associated genetic mutations may provide a tractable model to begin disentangling these possibilities, through behavioral assays and direct manipulations of brain function needed to fractionate the different components of reward (Berridge et al., 2009).

### Neural mechanisms for common and distinct dimensions of dysfunction

In light of the putative behavioral dimensions that are commonly impaired in ASDs and addiction, an obvious question arises regarding the elements of striatal circuitry that could commonly contribute to both disorders. One intriguing possibility involves D1-MSNs in the nucleus accumbens, as these cells appear to be a key locus for structural and functional synaptic changes caused by chronic drug exposure (Grueter et al., 2012; Smith et al., 2013). Drug-evoked plasticity at excitatory synapses on D1-MSNs in the nucleus accumbens appears to drive multiple forms of addiction-related behavior (Pascoli et al., 2012, 2014). Furthermore, direct activation of D1-MSNs in the nucleus accumbens enhances both drug reward and the development of psychomotor sensitization (Lobo et al., 2010; Koo et al., 2014), whereas inhibition of these cells reduces the same behavioral effects (Hikida et al., 2010; Ferguson et al., 2011).

Given this established link between addiction and D1-MSNs in the nucleus accumbens, it was both surprising and fascinating that recent reports linked these same cells to social behavior in wild-type mice (Gunaydin et al., 2014), as well as repetitive behavior in mice carrying ASD-associated mutations in *Nlgn3* (Rothwell et al., 2014). The former study suggests dopamine release and activation of D1-MSNs are both important for the expression of social behavior in mice (Gunaydin et al., 2014), whereas the latter study suggests synaptic disinhibition of D1-MSNs leads to repetitive behavior (Rothwell et al., 2014). These two patterns of results may appear contradictory in relation to ASDs, which are associated with less social behavior and more repetitive behavior. One potential explanation for this apparent contradiction (considered below) also provides insight into how the altered activity of one cell type could lead to both common and distinct dimensions of dysfunction.

Continuous synaptic disinhibition of D1-MSNs may lead to chronic elevation of baseline activity in these cells, thereby occluding phasic increases in activity that encode normal social behavior. Chronic elevation of baseline activity in D1-MSNs may also influence dopamine levels in the striatum, as axonal projections from these cells to the ventral tegmental area appear to synapse preferentially onto interneurons, which in turn inhibit dopamine neurons (Xia et al., 2011; Bocklisch et al., 2013). Disinhibition of D1-MSNs may therefore translate into disinhibition of dopamine neurons and increase dopamine release in the back into the striatum. This continuous increase in dopamine release would resemble the effects of ASD-associated genetic mutations in *Cntnap4* (Karayannis et al., 2014), and could lead to indiscriminate attribution of incentive salience to inappropriate stimuli in the environment. This “non-specific” attribution of incentive salience would be fundamentally different from the attribution of incentive salience in addiction, which is excessively large in magnitude but still specifically attributed to drugs and drug-related cues. Thus, increased activation of D1-MSNs and excessive dopamine release may be a common feature of ASDs and addiction, but there may be differences in how this cellular change is manifested in the clinical features of each disorder.

It is also becoming increasingly apparent that D1-MSNs in the nucleus accumbens exhibit heterogeneity in terms of function, structure, and neurochemistry. D1-MSNs specifically co-express the neuropeptide dynorphin, and activation of dynorphinergic cells in different subregions of the nucleus accumbens can lead to opposite behavioral effects (Al-Hasani et al., 2015). D1-MSNs in the nucleus accumbens also send axonal projections to both the ventral mesencephalon and ventral pallidum, and therefore contribute to both the direct and indirect pathways through the basal ganglia (Kupchik et al., 2015). Furthermore, a small population of MSNs in the nucleus accumbens appears to express both D1 and D2 dopamine receptor subtypes (Perreault et al., 2011). Subtle differences in how ASDs and addiction impact different subpopulations of D1-MSNs could also contribute to distinct clinical manifestations of these disorders.

### Therapeutic implications

Parallel dissection of striatal circuitry in ASDs and addiction may enable therapeutic advances in one field to inform progress in the other. For example, in the realm of addiction, the key role for D1-MSNs in promoting the behavioral effects of cocaine recently inspired an intersectional approach to reversing cocaine-evoked synaptic plasticity in mice, through the combination of deep brain stimulation of the nucleus accumbens and a pharmacological manipulation that selectively targets D1-MSNs (Creed et al., 2015). This type of intersectional strategy may prove beneficial as deep brain stimulation and other forms of neuromodulation are developed for clinical treatment of patients with ASDs (Sturm et al., 2012; Enticott et al., 2014; Sinha et al., 2015). Other types of intersectional therapeutic strategies could involve simultaneous manipulation of multiple neuromodulatory systems that intersect in the nucleus accumbens, such as oxytocin and serotonin (Dölen et al., 2013), or opioids and cannabinoids (Befort, 2015). The opioid and cannabinoid systems may be particularly tractable therapeutic targets, given their rich and diverse pharmacology as well as active drug development efforts for treatment of pain.

ASDs and drug addiction are complicated disorders that likely involve many parts of the brain, but the literature reviewed here highlights a central role for the striatum and basal ganglia in both disorders. In the striatum, these disorders impact a variety of neuromodulatory systems converging on multiple postsynaptic cells types. This intrinsic complexity makes it challenging to study striatal circuits in the context of disease, but also increases the number of potential therapeutic targets as well as the possibility of developing interventions that specifically affect individual circuit elements. It is quite likely that some common elements of this circuitry (like D1-MSNs in the nucleus accumbens) contribute to the pathophysiology of both ASDs and addiction, while other elements within or beyond the striatum are uniquely involved in only one disorder. A growing knowledge of both common and distinct dimensions of dysfunction will help guide the development of interventions that could be broadly useful for normalizing neural circuit dysfunction that contributes to behavioral deficits in both ASDs and addiction.

## Author contributions

The author confirms being the sole contributor of this work and approved it for publication.

### Conflict of interest statement

The author declares that the research was conducted in the absence of any commercial or financial relationships that could be construed as a potential conflict of interest.

## References

[B1] Abrams D. A. Lynch C. J. Cheng K. M. Phillips J. Supekar K. Ryali S. (2013). Underconnectivity between voice-selective cortex and reward circuitry in children with autism. Proc. Natl. Acad. Sci. U.S.A. 110, 12060–12065. 10.1073/pnas.1302982110 23776244 PMC3718181

[B2] Al-Hasani R. McCall J. G. Shin G. Gomez A. M. Schmitz G. P. Bernardi J. M.. (2015). Distinct subpopulations of nucleus accumbens dynorphin neurons drive aversion and reward. Neuron 87, 1063–1077. 10.1016/j.neuron.2015.08.019 26335648 PMC4625385

[B3] Albin R. L. Young A. B. Penney J. B. (1989). The functional anatomy of basal ganglia disorders. Trends Neurosci. 12, 366–375. 10.1016/0166-2236(89)90074-X 2479133

[B4] Amir R. E. Van Den Veyver I. B. Wan M. Tran C. Q. Francke U. Zoghbi H. Y. (1999). Rett syndrome is caused by mutations in X-linked MECP2, encoding methyl-CpG-binding protein 2. Nat. Genet. 23, 185–188. 10.1038/13810 10508514

[B5] Anacker A. M. Beery A. K. (2013). Life in groups: the roles of oxytocin in mammalian sociality. Front. Behav. Neurosci. 7:185. 10.3389/fnbeh.2013.00185 24376404 PMC3858648

[B6] Auerbach B. D. Osterweil E. K. Bear M. F. (2011). Mutations causing syndromic autism define an axis of synaptic pathophysiology. Nature 480, 63–68. 10.1038/nature10658 22113615 PMC3228874

[B7] Balleine B. W. Delgado M. R. Hikosaka O. (2007). The role of the dorsal striatum in reward and decision-making. J. Neurosci. 27, 8161–8165. 10.1523/JNEUROSCI.1554-07.2007 17670959 PMC6673072

[B8] Balleine B. W. O'Doherty J. P. (2010). Human and rodent homologies in action control: corticostriatal determinants of goal-directed and habitual action. Neuropsychopharmacology 35, 48–69. 10.1038/npp.2009.131 19776734 PMC3055420

[B9] Becker J. A. Clesse D. Spiegelhalter C. Schwab Y. Le Merrer J. Kieffer B. L. (2014). Autistic-like syndrome in mu opioid receptor null mice is relieved by facilitated mGluR4 activity. Neuropsychopharmacology 39, 2049–2060. 10.1038/npp.2014.59 24619243 PMC4104328

[B10] Befort K. (2015). Interactions of the opioid and cannabinoid systems in reward: insights from knockout studies. Front. Pharmacol. 6:6. 10.3389/fphar.2015.00006 25698968 PMC4318341

[B11] Belin D. Everitt B. J. (2008). Cocaine seeking habits depend upon dopamine-dependent serial connectivity linking the ventral with the dorsal striatum. Neuron 57, 432–441. 10.1016/j.neuron.2007.12.019 18255035

[B12] Berridge K. C. (2007). The debate over dopamine's role in reward: the case for incentive salience. Psychopharmacology (Berl.) 191, 391–431. 10.1007/s00213-006-0578-x 17072591

[B13] Berridge K. C. Robinson T. E. Aldridge J. W. (2009). Dissecting components of reward: ‘liking’, ‘wanting’, and learning. Curr. Opin. Pharmacol. 9, 65–73. 10.1016/j.coph.2008.12.014 19162544 PMC2756052

[B14] Bock R. Shin J. H. Kaplan A. R. Dobi A. Markey E. Kramer P. F.. (2013). Strengthening the accumbal indirect pathway promotes resilience to compulsive cocaine use. Nat. Neurosci. 16, 632–638. 10.1038/nn.3369 23542690 PMC3637872

[B15] Bocklisch C. Pascoli V. Wong J. C. House D. R. Yvon C. De Roo M.. (2013). Cocaine disinhibits dopamine neurons by potentiation of GABA transmission in the ventral tegmental area. Science 341, 1521–1525. 10.1126/science.1237059 24072923

[B16] Bowton E. Saunders C. Reddy I. A. Campbell N. G. Hamilton P. J. Henry L. K.. (2014). SLC6A3 coding variant Ala559Val found in two autism probands alters dopamine transporter function and trafficking. Transl. Psychiatry 4, e464. 10.1038/tp.2014.90 25313507 PMC4350523

[B17] Burguière E. Monteiro P. Mallet L. Feng G. Graybiel A. M. (2015). Striatal circuits, habits, and implications for obsessive-compulsive disorder. Curr. Opin. Neurobiol. 30, 59–65. 10.1016/j.conb.2014.08.008 25241072 PMC4293232

[B18] Burkett J. P. Spiegel L. L. Inoue K. Murphy A. Z. Young L. J. (2011). Activation of mu-opioid receptors in the dorsal striatum is necessary for adult social attachment in monogamous prairie voles. Neuropsychopharmacology 36, 2200–2210. 10.1038/npp.2011.117 21734650 PMC3176565

[B19] Burkett J. P. Young L. J. (2012). The behavioral, anatomical and pharmacological parallels between social attachment, love and addiction. Psychopharmacology (Berl.) 224, 1–26. 10.1007/s00213-012-2794-x 22885871 PMC3469771

[B20] Burkett Z. D. Day N. F. Peñagarikano O. Geschwind D. H. White S. A. (2015). VoICE: a semi-automated pipeline for standardizing vocal analysis across models. Sci. Rep. 5, 10237. 10.1038/srep10237 26018425 PMC4446892

[B21] Carlezon W. A. Jr. Thomas M. J. (2009). Biological substrates of reward and aversion: a nucleus accumbens activity hypothesis. Neuropharmacology 56(Suppl. 1), 122–132. 10.1016/j.neuropharm.2008.06.075 18675281 PMC2635333

[B22] Carp S. B. Rothwell E. S. Bourdon A. Freeman S. M. Ferrer E. Bales K. L. (2015). Development of a partner preference test that differentiates between established pair bonds and other relationships in socially monogamous titi monkeys (*Callicebus cupreus*). Am. J. Primatol. [Epub ahead of print]. 10.1002/ajp.22450 26235811 PMC5659711

[B23] Carter C. S. Devries A. C. Getz L. L. (1995). Physiological substrates of mammalian monogamy: the prairie vole model. Neurosci. Biobehav. Rev. 19, 303–314. 10.1016/0149-7634(94)00070-H 7630584

[B24] Centonze D. Rossi S. Mercaldo V. Napoli I. Ciotti M. T. De Chiara V.. (2008). Abnormal striatal GABA transmission in the mouse model for the fragile X syndrome. Biol. Psychiatry 63, 963–973. 10.1016/j.biopsych.2007.09.008 18028882

[B25] Chang J. Gilman S. R. Chiang A. H. Sanders S. J. Vitkup D. (2015). Genotype to phenotype relationships in autism spectrum disorders. Nat. Neurosci. 18, 191–198. 10.1038/nn.3907 25531569 PMC4397214

[B26] Chen J. A. Peñagarikano O. Belgard T. G. Swarup V. Geschwind D. H. (2015). The emerging picture of autism spectrum disorder: genetics and pathology. Annu. Rev. Pathol. 10, 111–144. 10.1146/annurev-pathol-012414-040405 25621659

[B27] Comings D. E. Comings B. G. Muhleman D. Dietz G. Shahbahrami B. Tast D.. (1991). The dopamine D2 receptor locus as a modifying gene in neuropsychiatric disorders. JAMA 266, 1793–1800. 10.1001/jama.1991.03470130073032 1832466

[B28] Creed M. Pascoli V. J. Luscher C. (2015). Addiction therapy. Refining deep brain stimulation to emulate optogenetic treatment of synaptic pathology. Science 347, 659–664. 10.1126/science.1260776 25657248

[B29] Curtis J. T. Liu Y. Aragona B. J. Wang Z. (2006). Dopamine and monogamy. Brain Res. 1126, 76–90. 10.1016/j.brainres.2006.07.126 16950234

[B30] De Krom M. Staal W. G. Ophoff R. A. Hendriks J. Buitelaar J. Franke B.. (2009). A common variant in DRD3 receptor is associated with autism spectrum disorder. Biol. Psychiatry 65, 625–630. 10.1016/j.biopsych.2008.09.035 19058789

[B31] Delmonte S. Balsters J. H. McGrath J. Fitzgerald J. Brennan S. Fagan A. J.. (2012). Social and monetary reward processing in autism spectrum disorders. Mol. Autism 3, 7. 10.1186/2040-2392-3-7 23014171 PMC3499449

[B32] DeLong M. R. (1990). Primate models of movement disorders of basal ganglia origin. Trends Neurosci. 13, 281–285. 10.1016/0166-2236(90)90110-V 1695404

[B33] Deng J. V. Rodriguiz R. M. Hutchinson A. N. Kim I. H. Wetsel W. C. West A. E. (2010). MeCP2 in the nucleus accumbens contributes to neural and behavioral responses to psychostimulants. Nat. Neurosci. 13, 1128–1136. 10.1038/nn.2614 20711186 PMC2928851

[B34] Deng J. V. Wan Y. Wang X. Cohen S. Wetsel W. C. Greenberg M. E.. (2014). MeCP2 phosphorylation limits psychostimulant-induced behavioral and neuronal plasticity. J. Neurosci. 34, 4519–4527. 10.1523/JNEUROSCI.2821-13.2014 24671997 PMC3965780

[B35] Di Martino A. Kelly C. Grzadzinski R. Zuo X. N. Mennes M. Mairena M. A.. (2011). Aberrant striatal functional connectivity in children with autism. Biol. Psychiatry 69, 847–856. 10.1016/j.biopsych.2010.10.029 21195388 PMC3091619

[B36] Dichter G. S. Felder J. N. Green S. R. Rittenberg A. M. Sasson N. J. Bodfish J. W. (2012). Reward circuitry function in autism spectrum disorders. Soc. Cogn. Affect. Neurosci. 7, 160–172. 10.1093/scan/nsq095 21148176 PMC3277365

[B37] Dölen G. Darvishzadeh A. Huang K. W. Malenka R. C. (2013). Social reward requires coordinated activity of nucleus accumbens oxytocin and serotonin. Nature 501, 179–184. 10.1038/nature12518 24025838 PMC4091761

[B38] Durieux P. F. Bearzatto B. Guiducci S. Buch T. Waisman A. Zoli M.. (2009). D2R striatopallidal neurons inhibit both locomotor and drug reward processes. Nat. Neurosci. 12, 393–395. 10.1038/nn.2286 19270687

[B39] Eliez S. Blasey C. M. Freund L. S. Hastie T. Reiss A. L. (2001). Brain anatomy, gender and IQ in children and adolescents with fragile X syndrome. Brain 124, 1610–1618. 10.1093/brain/124.8.1610 11459752

[B40] Enticott P. G. Fitzgibbon B. M. Kennedy H. A. Arnold S. L. Elliot D. Peachey A.. (2014). A double-blind, randomized trial of deep repetitive transcranial magnetic stimulation (rTMS) for autism spectrum disorder. Brain Stimul. 7, 206–211. 10.1016/j.brs.2013.10.004 24280031

[B41] Etherton M. Földy C. Sharma M. Tabuchi K. Liu X. Shamloo M.. (2011). Autism-linked neuroligin-3 R451C mutation differentially alters hippocampal and cortical synaptic function. Proc. Natl. Acad. Sci. U.S.A. 108, 13764–13769. 10.1073/pnas.1111093108 21808020 PMC3158170

[B42] Etherton M. R. Blaiss C. A. Powell C. M. Sudhof T. C. (2009). Mouse neurexin-1alpha deletion causes correlated electrophysiological and behavioral changes consistent with cognitive impairments. Proc. Natl. Acad. Sci. U.S.A. 106, 17998–18003. 10.1073/pnas.0910297106 19822762 PMC2764944

[B43] Everitt B. J. Robbins T. W. (2016). Drug addiction: updating actions to habits to compulsions ten years on. Annu. Rev. Psychol. 67, 23–50. 10.1146/annurev-psych-122414-033457 26253543

[B44] Ferguson S. M. Eskenazi D. Ishikawa M. Wanat M. J. Phillips P. E. Dong Y.. (2011). Transient neuronal inhibition reveals opposing roles of indirect and direct pathways in sensitization. Nat. Neurosci. 14, 22–24. 10.1038/nn.2703 21131952 PMC3058296

[B45] Földy C. Malenka R. C. Sudhof T. C. (2013). Autism-associated neuroligin-3 mutations commonly disrupt tonic endocannabinoid signaling. Neuron 78, 498–509. 10.1016/j.neuron.2013.02.036 23583622 PMC3663050

[B46] Fourgeaud L. Mato S. Bouchet D. Hémar A. Worley P. F. Manzoni O. J. (2004). A single *in vivo* exposure to cocaine abolishes endocannabinoid-mediated long-term depression in the nucleus accumbens. J. Neurosci. 24, 6939–6945. 10.1523/JNEUROSCI.0671-04.2004 15295029 PMC6729592

[B47] Fowler S. C. Birkestrand B. R. Chen R. Moss S. J. Vorontsova E. Wang G.. (2001). A force-plate actometer for quantitating rodent behaviors: illustrative data on locomotion, rotation, spatial patterning, stereotypies, and tremor. J. Neurosci. Methods 107, 107–124. 10.1016/S0165-0270(01)00359-4 11389948

[B48] Fuccillo M. V. Rothwell P. E. Malenka R. C. (2016). From synapses to behavior: what rodent models can tell us about neuropsychiatric disease. Biol. Psychiatry 79, 4–6. 10.1016/j.biopsych.2015.02.009 26616432 PMC4832414

[B49] Gerfen C. R. Surmeier D. J. (2011). Modulation of striatal projection systems by dopamine. Annu. Rev. Neurosci. 34, 441–466. 10.1146/annurev-neuro-061010-113641 21469956 PMC3487690

[B50] Gigliucci V. Leonzino M. Busnelli M. Luchetti A. Palladino V. S. D'Amato F. R.. (2014). Region specific up-regulation of oxytocin receptors in the opioid oprm1 (-/-) mouse model of autism. Front. Pediatr. 2:91. 10.3389/fped.2014.00091 25225634 PMC4150055

[B51] Gong S. Doughty M. Harbaugh C. R. Cummins A. Hatten M. E. Heintz N.. (2007). Targeting Cre recombinase to specific neuron populations with bacterial artificial chromosome constructs. J. Neurosci. 27, 9817–9823. 10.1523/JNEUROSCI.2707-07.2007 17855595 PMC6672645

[B52] Gong S. Zheng C. Doughty M. L. Losos K. Didkovsky N. Schambra U. B.. (2003). A gene expression atlas of the central nervous system based on bacterial artificial chromosomes. Nature 425, 917–925. 10.1038/nature02033 14586460

[B53] Graybiel A. M. (2008). Habits, rituals, and the evaluative brain. Annu. Rev. Neurosci. 31, 359–387. 10.1146/annurev.neuro.29.051605.112851 18558860

[B54] Grueter B. A. Brasnjo G. Malenka R. C. (2010). Postsynaptic TRPV1 triggers cell type-specific long-term depression in the nucleus accumbens. Nat. Neurosci. 13, 1519–1525. 10.1038/nn.2685 21076424 PMC3092590

[B55] Grueter B. A. Rothwell P. E. Malenka R. C. (2012). Integrating synaptic plasticity and striatal circuit function in addiction. Curr. Opin. Neurobiol. 22, 545–551. 10.1016/j.conb.2011.09.009 22000687 PMC3276730

[B56] Gunaydin L. A. Grosenick L. Finkelstein J. C. Kauvar I. V. Fenno L. E. Adhikari A.. (2014). Natural neural projection dynamics underlying social behavior. Cell 157, 1535–1551. 10.1016/j.cell.2014.05.017 24949967 PMC4123133

[B57] Haber S. N. Fudge J. L. McFarland N. R. (2000). Striatonigrostriatal pathways in primates form an ascending spiral from the shell to the dorsolateral striatum. J. Neurosci. 20, 2369–2382. 10704511 10.1523/JNEUROSCI.20-06-02369.2000 PMC6772499

[B58] Hamilton P. J. Campbell N. G. Sharma S. Erreger K. Herborg Hansen F. Saunders C.. (2013). *De novo* mutation in the dopamine transporter gene associates dopamine dysfunction with autism spectrum disorder. Mol. Psychiatry 18, 1315–1323. 10.1038/mp.2013.102 23979605 PMC4046646

[B59] Haznedar M. M. Buchsbaum M. S. Hazlett E. A. Licalzi E. M. Cartwright C. Hollander E. (2006). Volumetric analysis and three-dimensional glucose metabolic mapping of the striatum and thalamus in patients with autism spectrum disorders. Am. J. Psychiatry 163, 1252–1263. 10.1176/ajp.2006.163.7.1252 16816232

[B60] Heintz N. (2001). BAC to the future: the use of bac transgenic mice for neuroscience research. Nat. Rev. Neurosci. 2, 861–870. 10.1038/35104049 11733793

[B61] Hettinger J. A. Liu X. Hudson M. L. Lee A. Cohen I. L. Michaelis R. C.. (2012). DRD2 and PPP1R1B (DARPP-32) polymorphisms independently confer increased risk for autism spectrum disorders and additively predict affected status in male-only affected sib-pair families. Behav. Brain Funct. 8, 19. 10.1186/1744-9081-8-19 22559203 PMC3479424

[B62] Hettinger J. A. Liu X. Schwartz C. E. Michaelis R. C. Holden J. J. (2008). A DRD1 haplotype is associated with risk for autism spectrum disorders in male-only affected sib-pair families. Am. J. Med. Genet. B Neuropsychiatr. Genet. 147B, 628–636. 10.1002/ajmg.b.30655 18205172

[B63] Hikida T. Kimura K. Wada N. Funabiki K. Nakanishi S. (2010). Distinct roles of synaptic transmission in direct and indirect striatal pathways to reward and aversive behavior. Neuron 66, 896–907. 10.1016/j.neuron.2010.05.011 20620875

[B64] Hollander E. Anagnostou E. Chaplin W. Esposito K. Haznedar M. M. Licalzi E.. (2005). Striatal volume on magnetic resonance imaging and repetitive behaviors in autism. Biol. Psychiatry 58, 226–232. 10.1016/j.biopsych.2005.03.040 15939406

[B65] Ikemoto S. (2007). Dopamine reward circuitry: two projection systems from the ventral midbrain to the nucleus accumbens-olfactory tubercle complex. Brain Res. Rev. 56, 27–78. 10.1016/j.brainresrev.2007.05.004 17574681 PMC2134972

[B66] Im H. I. Hollander J. A. Bali P. Kenny P. J. (2010). MeCP2 controls BDNF expression and cocaine intake through homeostatic interactions with microRNA-212. Nat. Neurosci. 13, 1120–1127. 10.1038/nn.2615 20711185 PMC2928848

[B67] Insel T. Cuthbert B. Garvey M. Heinssen R. Pine D. S. Quinn K.. (2010). Research domain criteria (RDoC): toward a new classification framework for research on mental disorders. Am. J. Psychiatry 167, 748–751. 10.1176/appi.ajp.2010.09091379 20595427

[B68] Insel T. R. (2003). Is social attachment an addictive disorder? Physiol. Behav. 79, 351–357. 10.1016/S0031-9384(03)00148-3 12954430

[B69] Insel T. R. Cuthbert B. N. (2015). Medicine. Brain disorders? Precisely. Science 348, 499–500. 10.1126/science.aab2358 25931539

[B70] Insel T. R. Young L. J. (2001). The neurobiology of attachment. Nat. Rev. Neurosci. 2, 129–136. 10.1038/35053579 11252992

[B71] Jiang Y. H. Ehlers M. D. (2013). Modeling autism by SHANK gene mutations in mice. Neuron 78, 8–27. 10.1016/j.neuron.2013.03.016 23583105 PMC3659167

[B72] Jung K. M. Sepers M. Henstridge C. M. Lassalle O. Neuhofer D. Martin H.. (2012). Uncoupling of the endocannabinoid signalling complex in a mouse model of fragile X syndrome. Nat. Commun. 3, 1080. 10.1038/ncomms2045 23011134 PMC3657999

[B73] Karayannis T. Au E. Patel J. C. Kruglikov I. Markx S. Delorme R.. (2014). Cntnap4 differentially contributes to GABAergic and dopaminergic synaptic transmission. Nature 511, 236–240. 10.1038/nature13248 24870235 PMC4281262

[B74] Kohls G. Schulte-Rüther M. Nehrkorn B. Müller K. Fink G. R. Kamp-Becker I.. (2013). Reward system dysfunction in autism spectrum disorders. Soc. Cogn. Affect. Neurosci. 8, 565–572. 10.1093/scan/nss033 22419119 PMC3682440

[B75] Koo J. W. Lobo M. K. Chaudhury D. Labonté B. Friedman A. Heller E.. (2014). Loss of BDNF signaling in D1R-expressing NAc neurons enhances morphine reward by reducing GABA inhibition. Neuropsychopharmacology 39, 2646–2653. 10.1038/npp.2014.118 24853771 PMC4207344

[B76] Koob G. F. Bloom F. E. (1988). Cellular and molecular mechanisms of drug dependence. Science 242, 715–723. 10.1126/science.2903550 2903550

[B77] Kupchik Y. M. Brown R. M. Heinsbroek J. A. Lobo M. K. Schwartz D. J. Kalivas P. W. (2015). Coding the direct/indirect pathways by D1 and D2 receptors is not valid for accumbens projections. Nat. Neurosci. 18, 1230–1232. 10.1038/nn.4068 26214370 PMC4551610

[B78] Kwon C. H. Luikart B. W. Powell C. M. Zhou J. Matheny S. A. Zhang W.. (2006). Pten regulates neuronal arborization and social interaction in mice. Neuron 50, 377–388. 10.1016/j.neuron.2006.03.023 16675393 PMC3902853

[B79] Langen M. Bos D. Noordermeer S. D. Nederveen H. Van Engeland H. Durston S. (2014). Changes in the development of striatum are involved in repetitive behavior in autism. Biol. Psychiatry 76, 405–411. 10.1016/j.biopsych.2013.08.013 24090791

[B80] Langen M. Durston S. Staal W. G. Palmen S. J. Van Engeland H. (2007). Caudate nucleus is enlarged in high-functioning medication-naive subjects with autism. Biol. Psychiatry 62, 262–266. 10.1016/j.biopsych.2006.09.040 17224135

[B81] Langen M. Leemans A. Johnston P. Ecker C. Daly E. Murphy C. M.. (2012). Fronto-striatal circuitry and inhibitory control in autism: findings from diffusion tensor imaging tractography. Cortex 48, 183–193. 10.1016/j.cortex.2011.05.018 21718979

[B82] Langen M. Schnack H. G. Nederveen H. Bos D. Lahuis B. E. De Jonge M. V.. (2009). Changes in the developmental trajectories of striatum in autism. Biol. Psychiatry 66, 327–333. 10.1016/j.biopsych.2009.03.017 19423078

[B83] Levitt J. G. O'Neill J. Blanton R. E. Smalley S. Fadale D. McCracken J. T.. (2003). Proton magnetic resonance spectroscopic imaging of the brain in childhood autism. Biol. Psychiatry 54, 1355–1366. 10.1016/S0006-3223(03)00688-7 14675799

[B84] Liu Y. Aragona B. J. Young K. A. Dietz D. M. Kabbaj M. Mazei-Robison M.. (2010). Nucleus accumbens dopamine mediates amphetamine-induced impairment of social bonding in a monogamous rodent species. Proc. Natl. Acad. Sci. U.S.A. 107, 1217–1222. 10.1073/pnas.0911998107 20080553 PMC2824263

[B85] Lobo M. K. Covington H. E. III Chaudhury D. Friedman A. K. Sun H. Damez-Werno D.. (2010). Cell type-specific loss of BDNF signaling mimics optogenetic control of cocaine reward. Science 330, 385–390. 10.1126/science.1188472 20947769 PMC3011229

[B86] Maccarrone M. Rossi S. Bari M. De Chiara V. Rapino C. Musella A.. (2010). Abnormal mGlu 5 receptor/endocannabinoid coupling in mice lacking FMRP and BC1 RNA. Neuropsychopharmacology 35, 1500–1509. 10.1038/npp.2010.19 20393458 PMC3055456

[B87] Matthes H. W. Maldonado R. Simonin F. Valverde O. Slowe S. Kitchen I.. (1996). Loss of morphine-induced analgesia, reward effect and withdrawal symptoms in mice lacking the mu-opioid-receptor gene. Nature 383, 819–823. 10.1038/383819a0 8893006

[B88] McCarroll S. A. Hyman S. E. (2013). Progress in the genetics of polygenic brain disorders: significant new challenges for neurobiology. Neuron 80, 578–587. 10.1016/j.neuron.2013.10.046 24183011 PMC4066986

[B89] Mendez M. A. Horder J. Myers J. Coghlan S. Stokes P. Erritzoe D. (2013). The brain GABA-benzodiazepine receptor alpha-5 subtype in autism spectrum disorder: a pilot [(11)C]Ro15-4513 positron emission tomography study. Neuropharmacology 68, 195–201. 10.1016/j.neuropharm.2012.04.008 22546616 PMC4489617

[B90] Moles A. Kieffer B. L. D'Amato F. R. (2004). Deficit in attachment behavior in mice lacking the mu-opioid receptor gene. Science 304, 1983–1986. 10.1126/science.1095943 15218152

[B91] Nakatani J. Tamada K. Hatanaka F. Ise S. Ohta H. Inoue K.. (2009). Abnormal behavior in a chromosome-engineered mouse model for human 15q11-13 duplication seen in autism. Cell 137, 1235–1246. 10.1016/j.cell.2009.04.024 19563756 PMC3710970

[B92] Nelson A. B. Kreitzer A. C. (2014). Reassessing models of basal ganglia function and dysfunction. Annu. Rev. Neurosci. 37, 117–135. 10.1146/annurev-neuro-071013-013916 25032493 PMC4416475

[B93] Nestler E. J. Hyman S. E. (2010). Animal models of neuropsychiatric disorders. Nat. Neurosci. 13, 1161–1169. 10.1038/nn.2647 20877280 PMC3750731

[B94] Neuhofer D. Henstridge C. M. Dudok B. Sepers M. Lassalle O. Katona I.. (2015). Functional and structural deficits at accumbens synapses in a mouse model of Fragile X. Front. Cell. Neurosci. 9:100. 10.3389/fncel.2015.00100 25859182 PMC4374460

[B95] Neves-Pereira M. Muller B. Massie D. Williams J. H. O'Brien P. C. Hughes A.. (2009). Deregulation of EIF4E: a novel mechanism for autism. J. Med. Genet. 46, 759–765. 10.1136/jmg.2009.066852 19556253

[B96] Olazábal D. E. Young L. J. (2006). Species and individual differences in juvenile female alloparental care are associated with oxytocin receptor density in the striatum and the lateral septum. Horm. Behav. 49, 681–687. 10.1016/j.yhbeh.2005.12.010 16442534

[B97] Panksepp J. B. Lahvis G. P. (2007). Social reward among juvenile mice. Genes Brain Behav. 6, 661–671. 10.1111/j.1601-183X.2006.00295.x 17212648 PMC2040181

[B98] Pascoli V. Terrier J. Espallergues J. Valjent E. O'Connor E. C. Luscher C. (2014). Contrasting forms of cocaine-evoked plasticity control components of relapse. Nature 509, 459–464. 10.1038/nature13257 24848058

[B99] Pascoli V. Turiault M. Lüscher C. (2012). Reversal of cocaine-evoked synaptic potentiation resets drug-induced adaptive behaviour. Nature 481, 71–75. 10.1038/nature10709 22158102

[B100] Peca J. Feliciano C. Ting J. T. Wang W. Wells M. F. Venkatraman T. N.. (2011). Shank3 mutant mice display autistic-like behaviours and striatal dysfunction. Nature 472, 437–442. 10.1038/nature09965 21423165 PMC3090611

[B101] Peñagarikano O. Abrahams B. S. Herman E. I. Winden K. D. Gdalyahu A. Dong H.. (2011). Absence of CNTNAP2 leads to epilepsy, neuronal migration abnormalities, and core autism-related deficits. Cell 147, 235–246. 10.1016/j.cell.2011.08.040 21962519 PMC3390029

[B102] Perreault M. L. Hasbi A. O'Dowd B. F. George S. R. (2011). The dopamine d1-d2 receptor heteromer in striatal medium spiny neurons: evidence for a third distinct neuronal pathway in Basal Ganglia. Front. Neuroanat. 5:31. 10.3389/fnana.2011.00031 21747759 PMC3130461

[B103] Pieretti M. Zhang F. P. Fu Y. H. Warren S. T. Oostra B. A. Caskey C. T.. (1991). Absence of expression of the FMR-1 gene in fragile X syndrome. Cell 66, 817–822. 10.1016/0092-8674(91)90125-I 1878973

[B104] Portmann T. Yang M. Mao R. Panagiotakos G. Ellegood J. Dolen G.. (2014). Behavioral abnormalities and circuit defects in the basal ganglia of a mouse model of 16p11.2 deletion syndrome. Cell Rep. 7, 1077–1092. 10.1016/j.celrep.2014.03.036 24794428 PMC4251471

[B105] Resendez S. L. Dome M. Gormley G. Franco D. Nevárez N. Hamid A. A.. (2013). mu-Opioid receptors within subregions of the striatum mediate pair bond formation through parallel yet distinct reward mechanisms. J. Neurosci. 33, 9140–9149. 10.1523/JNEUROSCI.4123-12.2013 23699524 PMC6705037

[B106] Richey J. A. Rittenberg A. Hughes L. Damiano C. R. Sabatino A. Miller S.. (2014). Common and distinct neural features of social and non-social reward processing in autism and social anxiety disorder. Soc. Cogn. Affect. Neurosci. 9, 367–377. 10.1093/scan/nss146 23223206 PMC3980795

[B107] Robinson T. E. Berridge K. C. (2008). Review. The incentive sensitization theory of addiction: some current issues. Philos. Trans. R. Soc. Lond. B. Biol. Sci. 363, 3137–3146. 10.1098/rstb.2008.0093 18640920 PMC2607325

[B108] Rojas D. C. Peterson E. Winterrowd E. Reite M. L. Rogers S. J. Tregellas J. R. (2006). Regional gray matter volumetric changes in autism associated with social and repetitive behavior symptoms. BMC Psychiatry 6:56. 10.1186/1471-244X-6-56 17166273 PMC1770914

[B109] Rothwell P. E. Fuccillo M. V. Maxeiner S. Hayton S. J. Gokce O. Lim B. K.. (2014). Autism-associated neuroligin-3 mutations commonly impair striatal circuits to boost repetitive behaviors. Cell 158, 198–212. 10.1016/j.cell.2014.04.045 24995986 PMC4120877

[B110] Russo S. J. Dietz D. M. Dumitriu D. Morrison J. H. Malenka R. C. Nestler E. J. (2010). The addicted synapse: mechanisms of synaptic and structural plasticity in nucleus accumbens. Trends Neurosci. 33, 267–276. 10.1016/j.tins.2010.02.002 20207024 PMC2891948

[B111] Salamone J. D. Correa M. (2012). The mysterious motivational functions of mesolimbic dopamine. Neuron 76, 470–485. 10.1016/j.neuron.2012.10.021 23141060 PMC4450094

[B112] Santini E. Huynh T. N. Macaskill A. F. Carter A. G. Pierre P. Ruggero D.. (2013). Exaggerated translation causes synaptic and behavioural aberrations associated with autism. Nature 493, 411–415. 10.1038/nature11782 23263185 PMC3548017

[B113] Sarnyai Z. Kovacs G. L. (1994). Role of oxytocin in the neuroadaptation to drugs of abuse. Psychoneuroendocrinology 19, 85–117. 10.1016/0306-4530(94)90062-0 9210215

[B114] Sarnyai Z. Kovács G. L. (2014). Oxytocin in learning and addiction: from early discoveries to the present. Pharmacol. Biochem. Behav. 119, 3–9. 10.1016/j.pbb.2013.11.019 24280016

[B115] Scott-Van Zeeland A. A. Dapretto M. Ghahremani D. G. Poldrack R. A. Bookheimer S. Y. (2010). Reward processing in autism. Autism Res. 3, 53–67. 10.1002/aur.122 20437601 PMC3076289

[B116] Sears L. L. Vest C. Mohamed S. Bailey J. Ranson B. J. Piven J. (1999). An MRI study of the basal ganglia in autism. Prog. Neuropsychopharmacol. Biol. Psychiatry 23, 613–624. 10.1016/S0278-5846(99)00020-2 10390720

[B117] Sesack S. R. Grace A. A. (2010). Cortico-Basal Ganglia reward network: microcircuitry. Neuropsychopharmacology 35, 27–47. 10.1038/npp.2009.93 19675534 PMC2879005

[B118] Shuen J. A. Chen M. Gloss B. Calakos N. (2008). Drd1a-tdTomato BAC transgenic mice for simultaneous visualization of medium spiny neurons in the direct and indirect pathways of the basal ganglia. J. Neurosci. 28, 2681–2685. 10.1523/JNEUROSCI.5492-07.2008 18337395 PMC6670676

[B119] Silk T. J. Rinehart N. Bradshaw J. L. Tonge B. Egan G. O'Boyle M. W.. (2006). Visuospatial processing and the function of prefrontal-parietal networks in autism spectrum disorders: a functional MRI study. Am. J. Psychiatry 163, 1440–1443. 10.1176/ajp.2006.163.8.1440 16877661

[B120] Silverman J. L. Yang M. Lord C. Crawley J. N. (2010). Behavioural phenotyping assays for mouse models of autism. Nat. Rev. Neurosci. 11, 490–502. 10.1038/nrn2851 20559336 PMC3087436

[B121] Sinha S. McGovern R. A. Sheth S. A. (2015). Deep brain stimulation for severe autism: from pathophysiology to procedure. Neurosurg. Focus 38, E3. 10.3171/2015.3.FOCUS1548 26030703

[B122] Smith L. N. Jedynak J. P. Fontenot M. R. Hale C. F. Dietz K. C. Taniguchi M.. (2014). Fragile X mental retardation protein regulates synaptic and behavioral plasticity to repeated cocaine administration. Neuron 82, 645–658. 10.1016/j.neuron.2014.03.028 24811383 PMC4052976

[B123] Smith R. J. Lobo M. K. Spencer S. Kalivas P. W. (2013). Cocaine-induced adaptations in D1 and D2 accumbens projection neurons (a dichotomy not necessarily synonymous with direct and indirect pathways). Curr. Opin. Neurobiol. 23, 546–552. 10.1016/j.conb.2013.01.026 23428656 PMC3681928

[B124] Spiegel I. Salomon D. Erne B. Schaeren-Wiemers N. Peles E. (2002). Caspr3 and caspr4, two novel members of the caspr family are expressed in the nervous system and interact with PDZ domains. Mol. Cell. Neurosci. 20, 283–297. 10.1006/mcne.2002.1110 12093160

[B125] Staal W. G. Langen M. Van Dijk S. Mensen V. T. Durston S. (2015). DRD3 gene and striatum in autism spectrum disorder. Br. J. Psychiatry 206, 431–432. 10.1192/bjp.bp.114.148973 25792691

[B126] Sturm V. Fricke O. Buhrle C. P. Lenartz D. Maarouf M. Treuer H.. (2012). DBS in the basolateral amygdala improves symptoms of autism and related self-injurious behavior: a case report and hypothesis on the pathogenesis of the disorder. Front. Hum. Neurosci. 6:341. 10.3389/fnhum.2012.00341 23346052 PMC3549527

[B127] Su S. H. Kao F. C. Huang Y. B. Liao W. (2015). MeCP2 in the rostral striatum maintains local dopamine content critical for psychomotor control. J. Neurosci. 35, 6209–6220. 10.1523/JNEUROSCI.4624-14.2015 25878291 PMC6605173

[B128] Südhof T. C. (2008). Neuroligins and neurexins link synaptic function to cognitive disease. Nature 455, 903–911. 10.1038/nature07456 18923512 PMC2673233

[B129] Takarae Y. Minshew N. J. Luna B. Sweeney J. A. (2007). Atypical involvement of frontostriatal systems during sensorimotor control in autism. Psychiatry Res. 156, 117–127. 10.1016/j.pscychresns.2007.03.008 17913474 PMC2180158

[B130] Trezza V. Damsteegt R. Achterberg E. J. Vanderschuren L. J. (2011). Nucleus accumbens mu-opioid receptors mediate social reward. J. Neurosci. 31, 6362–6370. 10.1523/JNEUROSCI.5492-10.2011 21525276 PMC3098965

[B131] Trezza V. Damsteegt R. Manduca A. Petrosino S. Van Kerkhof L. W. Pasterkamp R. J.. (2012). Endocannabinoids in amygdala and nucleus accumbens mediate social play reward in adolescent rats. J. Neurosci. 32, 14899–14908. 10.1523/JNEUROSCI.0114-12.2012 23100412 PMC3496852

[B132] Trezza V. Damsteegt R. Vanderschuren L. J. (2009). Conditioned place preference induced by social play behavior: parametrics, extinction, reinstatement and disruption by methylphenidate. Eur. Neuropsychopharmacol. 19, 659–669. 10.1016/j.euroneuro.2009.03.006 19427175 PMC2716414

[B133] Turner K. C. Frost L. Linsenbardt D. McIlroy J. R. Müller R. A. (2006). Atypically diffuse functional connectivity between caudate nuclei and cerebral cortex in autism. Behav. Brain Funct. 2, 34. 10.1186/1744-9081-2-34 17042953 PMC1635430

[B134] Tzschentke T. M. (1998). Measuring reward with the conditioned place preference paradigm: a comprehensive review of drug effects, recent progress and new issues. Prog. Neurobiol. 56, 613–672. 10.1016/S0301-0082(98)00060-4 9871940

[B135] Vanderschuren L. J. Kalivas P. W. (2000). Alterations in dopaminergic and glutamatergic transmission in the induction and expression of behavioral sensitization: a critical review of preclinical studies. Psychopharmacology (Berl.) 151, 99–120. 10.1007/s002130000493 10972458

[B136] Voelbel G. T. Bates M. E. Buckman J. F. Pandina G. Hendren R. L. (2006). Caudate nucleus volume and cognitive performance: are they related in childhood psychopathology? Biol. Psychiatry 60, 942–950. 10.1016/j.biopsych.2006.03.071 16950212 PMC2947855

[B137] Wang H. Wu L. J. Kim S. S. Lee F. J. Gong B. Toyoda H.. (2008). FMRP acts as a key messenger for dopamine modulation in the forebrain. Neuron 59, 634–647. 10.1016/j.neuron.2008.06.027 18760699

[B138] Wei D. Lee D. Cox C. D. Karsten C. A. Penagarikano O. Geschwind D. H.. (2015). Endocannabinoid signaling mediates oxytocin-driven social reward. Proc. Natl. Acad. Sci. U.S.A. 112, 14084–14089. 10.1073/pnas.1509795112 26504214 PMC4653148

[B139] Weiss L. A. Shen Y. Korn J. M. Arking D. E. Miller D. T. Fossdal R.. (2008). Association between microdeletion and microduplication at 16p11.2 and autism. N. Engl. J. Med. 358, 667–675. 10.1056/NEJMoa075974 18184952

[B140] Whittemore R. Chao A. Jang M. Minges K. E. Park C. (2014). Methods for knowledge synthesis: an overview. Heart Lung 43, 453–461. 10.1016/j.hrtlng.2014.05.014 25012634

[B141] Wise R. A. (1987). The role of reward pathways in the development of drug dependence. Pharmacol. Ther. 35, 227–263. 10.1016/0163-7258(87)90108-2 3321101

[B142] Wise R. A. (2004). Dopamine, learning and motivation. Nat. Rev. Neurosci. 5, 483–494. 10.1038/nrn1406 15152198

[B143] Xia Y. Driscoll J. R. Wilbrecht L. Margolis E. B. Fields H. L. Hjelmstad G. O. (2011). Nucleus accumbens medium spiny neurons target non-dopaminergic neurons in the ventral tegmental area. J. Neurosci. 31, 7811–7816. 10.1523/JNEUROSCI.1504-11.2011 21613494 PMC6633124

[B144] Yamasue H. Yee J. R. Hurlemann R. Rilling J. K. Chen F. S. Meyer-Lindenberg A.. (2012). Integrative approaches utilizing oxytocin to enhance prosocial behavior: from animal and human social behavior to autistic social dysfunction. J. Neurosci. 32, 14109–14117. 10.1523/JNEUROSCI.3327-12.2012 23055480 PMC6622380

[B145] Yin H. H. Knowlton B. J. (2006). The role of the basal ganglia in habit formation. Nat. Rev. Neurosci. 7, 464–476. 10.1038/nrn1919 16715055

[B146] Young K. A. Liu Y. Gobrogge K. L. Wang H. Wang Z. (2014). Oxytocin reverses amphetamine-induced deficits in social bonding: evidence for an interaction with nucleus accumbens dopamine. J. Neurosci. 34, 8499–8506. 10.1523/JNEUROSCI.4275-13.2014 24948805 PMC4061391

[B147] Zoghbi H. Y. Bear M. F. (2012). Synaptic dysfunction in neurodevelopmental disorders associated with autism and intellectual disabilities. Cold Spring Harb. Perspect. Biol. 4:a009886. 10.1101/cshperspect.a009886 22258914 PMC3282414

